# Stress-induced hyperphagia: empirical characterization of stress-overeaters

**DOI:** 10.1186/s12889-021-12488-9

**Published:** 2022-01-14

**Authors:** Birgit Kaiser, Kathrin Gemesi, Sophie Laura Holzmann, Monika Wintergerst, Martin Lurz, Hans Hauner, Georg Groh, Markus Böhm, Helmut Krcmar, Christina Holzapfel, Kurt Gedrich

**Affiliations:** 1 Research Group Public Health Nutrition, ZIEL - Institute for Food & Health, Technical University of Munich, Weihenstephaner Berg 1, 85354 Freising, Germany; 2 Institute for Nutritional Medicine, School of Medicine, Technical University of Munich, Munich, Germany; 3 Research Group Social Computing, Department of Informatics, Technical University of Munich, Garching, Germany; 4 Krcmar Lab, Department of Informatics, Technical University of Munich, Garching, Germany

**Keywords:** Stress, Nutrition, Overeating, Coping, Eating motives, Personality

## Abstract

**Background:**

Stressful situations can have an impact on an individual’s eating behavior. People vulnerable to the influence of stress tend to change the quantity and quality of their food intake. Variables such as sex and body mass index (BMI) seem to be related to this stress-eating behavior, but it is rather unclear what factors account to the parameters associated with stress-eating behavior. The aim of this survey was to identify further characteristics of adults in Germany related to stress-overeating, focusing on stress perception, coping, eating motives and comfort foods as well as personality types.

**Methods:**

This online survey was performed throughout Germany and comprised a 38-item pre-tested questionnaire. Stress-induced overeating was classified based on the Salzburg Stress Eating Scale (SSES). Moreover, validated questionnaires were used to identify additional characteristics of stress eaters. Participants were recruited using a convenience sampling approach, and data were collected between January and April 2021.

**Results:**

The overall sample consisted of 1222 participants (female 80.8%, aged 31.5±12.8). 42.1% of participants were identified as stress-overeaters. Among the remaining group, 78.9% stated to eat less, 21.1% to eat equally when stressed. Female participants had a higher mean SSES score compared to male participants. The BMI was positively correlated to SSES, r(1220)=0.28, *p>0.005*. ‘Agreeableness’ (BigFive) was found to be a negative predictor of stress-overeating. The most pronounced difference in eating motives (The Eating Motivation Survey, TEMS) was found for ‘Affect Regulation’ and ‘Weight Control’.

**Conclusions:**

The results indicate that stress-overeating affects a large proportion of the surveyed population. BMI, personality and eating motives additionally characterize stress-overeaters and may contribute to develop new approaches to address unhealthy stress-related eating patterns.

## Background

Unhealthy dietary patterns are still a main risk factor for the development of non-communicable diseases, accounting for 11 million deaths and 255 million disability-adjusted life years (DALYS) globally [1]. Especially, a high intake of sodium as well as insufficient intakes of whole grains fiber and fruit were found to be leading dietary risk factors. Unhealthy dietary patterns (e.g., energy-dense food) particularly contribute to the development of obesity, type 2 diabetes and the metabolic syndrome [2]. Stress is one risk factors for the development of unhealthy eating habits (e.g., binge eating or overeating), which can lead to serious health problems like overweight and obesity [3, 4].

Lazarus and Folkman (1984, p.19) defined stress as “a particular relationship between the person and the environment that is appraised by the person as taxing or exceeding his or her resources and endangering his or her well-being” [5]. Different mechanisms to compensate stress are activated to maintain the homeostasis of the organism. Stress triggers the autonomic nervous system (ANS), signaling alert, to prepare the organism for a fight or flight reaction by ,e.g., increasing the heart rate and releasing stress hormones [6]. The activation of the hypothalamic pituitary adrenal (HPA) triggers the release of corticotropin releasing hormone (CRH), which mediates the suppression of food intake. The CRH stimulates the release of adrenocorticotropic hormone, which contributes to the release of glucocorticoid hormones, e.g., cortisol. After less severe stress, increased levels of glucocorticoids and cortisol are associated with increased appetite [7].

It was shown that 82% of a population change the amount of their food intake in the context of stress [8]. People who eat more when stressed are defined as hyperphagic and people who eat less when stressed are defined as hypophagic [9]. Besides the general increase or decrease of food quantities due to the experience of stress, research focuses on the quality of the consumed foods. Stress tends to stimulate the sensitivity of the reward system, leading to craving for hyperpalatable or comfort foods [10]. Additionally, the prioritization of eating motives changes and the eating behavior is shifted to emotional instead of nutritional needs [11]. Stress-related changes in eating behavior are associated with various factors. Regarding anthropometrics, perceived stress has been found to be positively correlated with short-term weight change (*r* = 0.35, *p* = 0.01) [12]. Forms of overeating due to perceived stress and negative affect have a higher prevalence in women than in men [13].

Additionally, emotion-based eating is mediated by individual differences in stress perception. Brouwer et al. [14] described the association of certain types of personality and stress sensitivity. To categorize personality types, the five-factor model is often used [15]. Whereas on the one hand, neuroticism is positively correlated with stress sensitivity, conscientiousness, on the other hand, is negatively correlated with stress sensitivity. Supplementary, certain types of personality are associated with emotional eating, specifically neuroticism, low conscientiousness and low extraversion [16].

Furthermore, adequate stress coping capabilities (e.g., social support) are seen to have a mediating effect on the perception of stress (e.g., depression) [17]. ‘Positive Thinking’, seeing the good side of stress, ‘Active Stress Coping’, being proactive to prevent stress, ‘Social Support’, having people around who help to manage stress and ‘Keeping Faith’, finding hold in faith, are adaptive coping strategies. ‘Increased Alcohol and Cigarette Consumption’, relying on alcohol and cigarettes if stress gets overwhelming, is categorized as maladaptive coping strategy [18]. Inadequate coping strategies, however, can lead to disordered eating behaviors [19]. Taken together, various aspects seem to influence the vulnerability of individuals to stress-induced unhealthy eating patterns.

Evidence on other relevant factors associated with overeating in the context of stress is still rare. Therefore, the aim of this study was to explore stress-eating behavior of people in Germany. In detail, this study focused on the identification of associations between stress-overeating behavior and 1) anthropometric variables, 2) perceived stress, 3) stress coping, 4) eating motives and behavior (including comfort foods) and 5) type of personality.

## Methods

### Study design and sample

This open online survey was conducted between January 6th and April 21st, 2021. Informed consent was obtained by the participants at the start of the online survey.

To meet the inclusion criteria, participants needed to be full of age (18+ years), able to read and write in German and sign the digital approval to declaration of consent and protection of privacy.

Recruitment was executed digitally throughout Germany. Homepages, mailing lists and social media accounts were used to display study information. Additionally, mailing lists and social media accounts of the involved institutes and further corporations were used to recruit participants. Potential study participants were guided to the survey with an online link.

### Survey development

The survey questionnaire was developed iteratively by an interdisciplinary team of nutritionists, public health experts and computer scientists. The preliminary version was pre-tested by a group of non-project members (*n* = 10) and modified (comprehensibility and length of survey) subsequently. The final questionnaire consisted of 38 items, including validated questionnaires and was designed using the SoSciSurvey survey platform (V3.1.06,[20]). Information about the research project, protection of data privacy and voluntary participation were integrated within the introductory section. The survey focused on five key aspects: eating behavior, stress (perception and coping), stress-eating, technical behavior (general technical innovativeness and IT adoption and acceptance of fictive digital stress application) and personality. Sociodemographic and self-reported anthropometric data were collected at the end of the survey. Most survey items were issued as short and easy to understand question items with closed, open, single or multiple-choice answering options. Filter questions were integrated to simplify the completion of the questionnaire. Items on technical behavior are beyond the scope of the current considerations and were therefore neglected for analysis. Five psychometrical tools and eight additional question items were appraised for primary analysis within this manuscript.

### Assessment of stress eating

Changes in the eating behavior in response to stressful events were appraised using the validated *Salzburg Stress Eating Scale* (SSES) [21]. Items are scored with 1-5, mean results categorize participants into three different response types: ‘eats less when stressed’ (score<3), ‘eats the same amount as usual’ (score=3) and ‘eats more when stressed’ (score>3) [21]. Additionally, we asked participants to subjectively evaluate their stress response and categorize themselves as stress-overeaters or non stress-overeaters.

### Assessment of perceived stress

Participants’ stress level was assessed using the validated *Perceived Stress Scale* (PSS) [22]. The PSS addresses stressful situations within the previous month and captures the frequency of specific feelings and thoughts within this period. The ten items are scored with 0-4. Individual total sum scores indicate the amount of perceived stress, higher scores indicating higher perceived stress. An additional question was integrated into the survey to capture the frequencies of stressful events within the last month, including answering options ranging from daily, a few times a week, a few times a month, seldom to never.

### Assessment of stress coping

Coping with stress was determined using the validated *Stress and Coping Inventory* (SCI) [18] which considers adaptive (‘Positive Thinking’, ‘Active Stress Coping’, ‘Social Support’, ‘Keeping Faith’) and maladaptive coping strategies (‘Increased Alcohol and Cigarette Consumption’). Items are scored between 1-4, resulting in a total sum score for each coping strategy scale. Higher scores indicate greater relevance of the respective coping strategy [18].

### Assessment of eating motives

We applied the validated *The Eating Motivation Survey* (TEMS) [23] to address the participants’ eating motives. It provides the relative importance of 15 eating motives such as ‘Liking’, ‘Habits’, ‘Price’, or ‘Visual Appeal’. Items are scored between 1 and 7, mean scores are calculated for each motive. Higher mean scores indicate greater relative importance of the respective motive [23].

### Assessment of comfort food consumption frequencies

Based on a literature search, the following 13 comfort foods (including beverages) were identified, as being preferably consumed in stressful situations: chocolate & confectionery, sweets, ice cream, cake, cookies, chips & crackers, salted nuts, fried food & fries, fast foods (burger, curry sausages or pizza), alcohol, sugar-sweetened beverages, energy drinks and coffee [24–26]. Participants were presented with this closed list of preselected comfort foods and asked to indicate the frequency of their consumption in stressful situations based on a 5-point Likert scale ranging from 0=never to 4=very often. Answers ‘often’ and ‘very’ often (3 and 4) were considered as positive value for the consumption of the comfort food and therefore dummy-coded with 1. Answers ‘sometimes’, ‘seldom’ and ‘never’ were dummy coded with 0.

### Categorization of personality

The *Big Five Inventory* (BFI-10) was used to assess the participants’ personalities [27]. Ten items, two for each dimension, were displayed and mean scores for each dimension were calculated. Scores ranged between 1-5, determining the importance of the five personality dimensions ‘Openness’, ‘Conscientiousness’, ‘Extraversion’, ‘Agreeableness’ and ‘Neuroticism’. The higher the score of a dimension, the more this dimension contributes to one’s personality [27].

### Anthropometric data

Sex, age, weight and height were addressed by self-reporting question items.

### Statistical analysis

At first, data were checked for integrity and plausibility. Respondents with missing or seemingly invalid data (e.g., BMI <17 kg/m^2^ or > 50 kg/m^2^) were excluded. The performed data analysis focused on items relevant for the possible association with stress-overeater characteristics. The identification of stress-overeaters was based on the SSES scores. Stress-eaters are defined as individuals who change their eating behavior due to stress situations, irrespective of the direction of change (hyper- as well as hypophagia) [28]. Accordingly, respondents who were categorized as ‘eats more’ when stressed according to the SSES valuation, are hereinafter referred to as stress-overeaters, respondents categorized as ‘eats less’ are referred to as stress-undereaters and participants categorized as ‘eats equal’ when stressed are referred to as stress-insensitive eaters. These three stress-eater subgroups were compared with respect to anthropometrics as well as PSS, SCI, TEMS, Big Five. Descriptive analysis (frequencies and percentages), non-parametric test for subgroup analysis (Kruskal–Wallis test and Wilcoxon signed-rank test) as well as inferential statistics (linear regression, z-standardized), were performed and corresponding effect sizes (eta squared and Cramer’s V [29]) estimated. Linear regression was performed as backward selection starting from a full model with all variables. Non-significant variables were neglected stepwise, resulting in a final reduced model. Adjusted R^2^ are reported to describe the goodness of fit. Data analyses were performed using Microsoft Excel 2016 (Microsoft Corp) and R 3.6.0 (R Foundation). *P*-values of *p≤0.05* were considered as indicating statistical significance.

## Results

### Characteristics of the survey population

In total, data from 1222 participants were included in the final analysis. Participants’ sociodemographic and anthropometric characteristics are presented in Table 1. The sample consisted of 80.8% women. Participants’ age ranged between 18 and 82 years (m=31.5, sd=12.8). The BMI range was between 17.1 kg/m^2^ and 48.4 kg/m^2^ (m=23.4 kg/m^2^, sd=4.3 kg/m^2^). Most of the participants were single (65.9%) and with a secondary school degree (88.4%). More than half of the participants were students (53.4%).

**Table 1:** Sociodemographic and anthropometric characteristics of participants

Characteristics	Absolute (%) of participants	Mean (±sd)
Total	1222 (100.0)	
Age groups		31.48 (±12.8)
18-29 years	770 (63.0)	
30-49 years	2690 (22.0)	
50-64 years	166 (13.6)	
≥65 years	17 (1.4)	
Sex		
Female	987 (80.8)	
Male	235 (19.2)	
BMI (kg/m^2^)		23.43 (±4.3)
Underweight (<18.5)	59 (4.8)	17.79 (±0.7)
Normal weight (18.5 - <25)	841 (68.8)	21.69 (±1.8)
Pre-obese (25 - <30)	227 (18.6)	26.90 (±1.4)
Obese (≥30)	95 (7.8)	34.01 (±4.3)
Level of education		
Less than secondary school degree	92 (7.5)	
Secondary school degree	1087 (89.1)	
School education ongoing	10 (0.8)	
Other	32 (2.6)	
Profession		
Apprentice	14 (1.1)	
Student	652 (53.4)	
Employee	428 (35.0)	
Self-employed	63 (5.2)	
Job applicant	14 (1.1)	
Not working (maternal leave, housewife/-man)	33 (2.7)	
Other	18 (1.5)	

### Stress eating behavior and perceived stress

Participants were categorized with respect to their stress-eating behavior according to their SSES scores (Table 2). 42.1% of the subjects were identified as stress-induced hyperphagic eaters. Besides the validated SSES, participants subjectively evaluated their stress-eating behavior. 48.5% of participants subjectively considered themselves as stress-overeaters, whereas 51.5% regarded themselves as non stress-overeater. A strong association was found between the SSES (stress-undereaters and stress-insensitive eaters have been summed up as non stress-overeaters) and the subjective classification of stress-induced overeating, X^2^(1, 1222)=488.05, *p<0.005*.

**Table 2:** Salzburg Stress Eating Scale (SSES)

Measurement	Absolut (%) of participants	Mean (sd)
**Salzburg Stress Eating Scale**		3.0 (±0.73)
Eat less when stressed (“stress-undereater”)	558 (45.7)	
Eat equally when stressed (“stress-insensitive eater”)	149 (12.2)	
Eat more when stressed (“stress-overeater”)	515 (42.1)	

Female participants had a higher SSES mean score (m=3.1, sd=0.8) compared to male participants (m=2.9, sd=0.6), *U=98659.0 p<0.0005*. Focusing on BMI, a positive correlation between BMI and SSES was determined, r(1220)=0.28, *p<0.005*. No statistically significant association between age and SSES was found (*p* = 0.60).

**Fig. 1:**
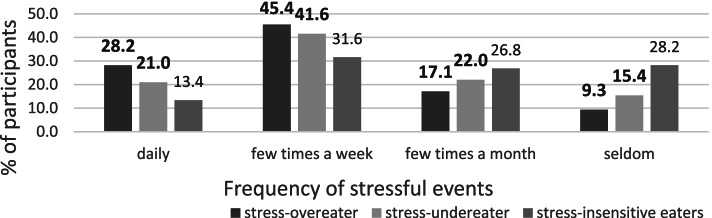
Frequency of stressful events in stress-overeaters compared to stress-undereaters and stress-insensitive eaters

Across all participants, a mean PSS score of 19.3 (sd=6.8) was found, representing a moderate stress level [30]. People with hyperphagia had a higher PSS score (m=20.8, sd=6.5) than people with hypophagia (m=18.7, sd=6.8) and stress-insensitive eaters (m=16.9, sd=7.1) *H(2)= 52.1, p<0.005*. The PSS and SSES scores were positively correlated r(1220)=0.14*, p<0.005*. Regarding the frequency of stressful events, most participants reported feeling stressed a few times a week (42.0%). 23.1% were stressed daily, 20.5% were stressed a few times a month and 14.4% reported to be seldomly stressed (Fig. 1). No participant stated to never be stressed. Between SSES and stress frequency, a weak association was found, X^2^(6,1222)=53.98, *p*<0.005, V=0.149.

### Stress coping

Complementary to perceived stress, the stress coping behavior was addressed. The usage of the SCI strategies is shown in Table 3.

**Table 3:** Usage of stress coping strategies (SCI)

Coping strategies	Total sample Mean (±sd)	Stress-overeater Mean (±sd)	Stress-undereater Mean (±sd)	Stress-insensitive eater Mean (±sd)	Mean difference between groups *p*-value*
Positive thinking	10.4 (±2.3)	10.2 (±1.3)	10.5 (±2.3)	10.6 (±2.3)	*0.06*
Active stress coping	10.7 (±2.3)	10.5 (±2.2)	10.8 (±2.3)	11.0 (±2.4)	*0.12*
Social support	12.8 (±2.7)	12.7 (±2.8)	12.9 (±2.9)	12.8 (±2.5)	*0.55*
Increased alcohol and cigarette consumption	7.4 (±1.2)	7.4 (±1.2)	7.3 (±1.2)	7.5 (±1.0)	*0.30*
Keeping faith	7.2 (±2.6)	7.2 (±2.6)	7.2 (±2.8)	7.0 (±2.6)	*0.54*

*p-values were calculated applying the Kruskal–Wallis test

Overall, the study participants achieve higher scores for adaptive coping strategies (‘Positive Thinking’, ‘Active Stress Coping’ and ‘Social Support’) than for maladaptive or religious ones. There were no significant differences between different stress-eating responders in the context of coping strategies.

### Eating behavior

Eating behavior was addressed using the TEMS. Table 4 presents the relative importance of eating motives.

**Table 4:** Importance of eating motives (TEMS)

Eating motivations	Total sample Mean (±sd)	Stress-overeater Mean (±sd)	Stress-undereaterMean (±sd)	Stress-insensitive eater Mean (±sd)	Mean difference between groups *p*-value*	Effect size η^2^**
Liking	6.2 (±0.9)	6.2 (±0.9)	6.3 (±0.9)	6.2 (±0.9)	*0.30*	
Need & hunger	5.5 (±1.2)	5.3 (±1.2)	5.6 (±1.2)	5.6 (±1.1)	*<0.005*	*0.013*
Health	5.2 (±1.2)	5.2 (±1.2)	5.2 (±1.2)	5.2 (±1.1)	*0.97*	
Habits	5.1 (±1.3)	5.3 (±1.2)	4.9 (±1.4)	5.1 (±1.3)	*<0.005*	*0.016*
Pleasure	4.9 (±1.3)	5.1 (±1.3)	4.8 (±1.4)	4.7 (±1.3)	*<0.005*	*0.02*
Convenience	4.4 (±1.5)	4.5 (±1.5)	4.3 (±1.5)	4.1 (±1.5)	*0.01*	*0.001*
Natural concerns	4.3 (±1.8)	4.3 (±1.8)	4.3 (±1.8)	4.3 (±1.8)	*0.97*	
Sociability	4.3 (±1.6)	4.4 (±1.6)	4.2 (±1.6)	4.1 (±1.7)	*0.03*	*0.004*
Weight control	4.0 (±1.7)	4.4 (±1.6)	3.7 (±1.8)	3.5 (±1.7)	*<0.005*	*0.035*
Traditional eating	3.7 (±1.7)	3.8 (±1.8)	3.6 (±1.7)	3.7 (±1.6)	*0.17*	
Visual appeal	3.6 (±1.6)	3.8 (±1.6)	3.5 (±1.6)	3.2 (±1.5)	*<0.005*	*0.016*
Price	3.4 (±1.6)	3.5 (±1.6)	3.3 (±1.5)	3.1 (±1.4)	*0.01*	*0.006*
Affect regulations	3.3 (±1.9)	4.4 (±1.7)	2.6 (±1.6)	2.4 (±1.6)	*<0.005*	*0.241*
Social norms	2.1 (±1.4)	2.3 (±1.5)	2.0 (±1.3)	1.9 (±1.3)	*<0.005*	*0.013*
Social image	1.9 (±1.2)	2.0 (±1.3)	1.8 (±1.2)	1.6 (±1.2)	*0.06*	

Effect benchmarks: small (η^2^=0.02), medium (η^2^=0.13), large (η^2^=0.26) [31]

*p-values were calculated applying the Kruskal–Wallis test

** η^2^ was only calculated for motives with sign. *p*-value,

On average, stress-overeaters had statistically significant higher importance scores in nine out of 15 motives. The greatest differences between stress-overeaters and non stress-overeaters were yielded for ‘Affect Regulation’ (stress-undereaters: Δ=1.8, *p<0.005;* stress-insensitive eaters: Δ=2.0, *p<0.005*) and ‘Weight Control’ (stress-undereaters: Δ=0.7, *p<0.005;* stress-insensitive eaters: Δ=0.9, *p<0.005*). On the contrary, non stress-overeaters achieved considerably higher importance scores for the motive of ‘Need & Hunger’ (stress-undereaters: Δ=0.3, *p<0.005;* stress-insensitive eaters: Δ=0.3, *p<0.005*).

### Comfort foods

**Fig. 2:**
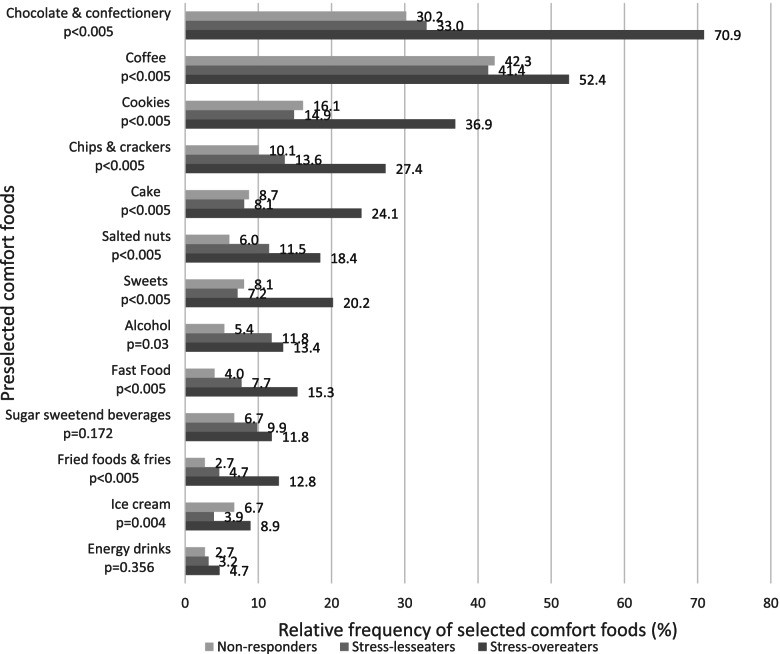
Relative consumption of selected comfort foods among stress-eater subgroups. *p-values were calculated applying X^*2*^-test

Figure 2 presents the frequency of comfort foods, which were consumed often or very often by the participants in the context of stress. Multiple comfort foods could be selected.

Participants selected (multiple) comfort foods, based on a closed list of 13 pre-selected comfort food options. On average, stress-overeaters selected 3.2 out of 13 comfort food items, whereas stress-undereaters choose a mean of 1.7 and stress-insensitive eaters a mean of 1.5 frequently consumed comfort food items. Most commonly, stress-overeaters chose chocolate & confectionery as comfort food (stress-overeaters 70.9%, stress-undereaters 33.0%, stress-insensitive eaters 30.2%, X^2^(2, 1222)=177.0, *p<0.005,* V=0.380 ), followed by coffee (stress-overeaters 52.4%, stress-undereaters 41.4%, stress-insensitive eaters 42.3%, X^2^(2, 1222)=14.1, *p<0.005*, V=0.108) and cookies (stress-overeaters 36.9%, stress-undereaters 14.9%, stress-insensitive eaters 16.1%, X^2^(2, 1222)=76.8, *p<0.005*, V=0.251).

### Personality

Personality dimensions were evaluated using the BigFive questionnaire. The importance of the dimensions are displayed in Table 5.

**Table 5:** Importance of personality dimensions (BigFive)

Personality dimensions	Total sample Mean (±sd)	Stress-overeater Mean (±sd)	Stress-undereater Mean (±sd)	Stress-insensitive eater Mean (±sd)	Mean difference between groups *p*-value*	Effect size η^2^**
Agreeableness	3.2 (±0.8)	3.2 (±0.8)	3.2 (±0.7)	3.2 (±0.8)	*0.83*	
Extraversion	3.2 (±0.1)	3.2 (±1.0)	3.2 (±1.0)	3.1 (±0.9)	*0.30*	
Conscientiousness	3.1 (±0.5)	3.1 (±0.5)	3.1 (±0.5)	3.1 (±0.5)	*0.48*	
Openness	2.8 (±0.6)	2.9 (±0.6)	2.9 (±0.6)	2.8 (±0.6)	*0.13*	
Neuroticism	2.7 (±0.6)	2.7 (±0.6)	2.7 (±0.6)	2.5 (±0.7)	*0.01*	0.006

Effect benchmarks: small (η^2^=0.02), medium (η^2^=0.13), large (η^2^=0.26) [31]

*p-values were calculated applying the Kruskal–Wallis test

** η^2^ was only calculated for motives with sign. *p*-value

Concerning their personality, stress-overeaters did not differ on average from non stress-overeaters, except for the dimension ‘Neuroticism’ where stress-overeaters scored significantly higher than stress-insensitive eaters (Δ=0.2, *p=0.01*, n^2^=0.006.

### SSES predicting model

We developed a multiple linear regression model regressing SSES on possible predictors such as sex, BMI, age, PSS sum score, strategies of stress coping inventory (SCI), the eating motivation survey (TEMS) and big five personality dimensions to control for confounders. Results of the adapted (backward selection) multiple linear regression model are presented in Table 6. BMI, ‘Agreeableness’ personality (BigFive) as well as ‘Habits’, ‘Health’, ‘Traditional Eating’, ‘Weight Control’ and ‘Affect Regulation’ (TEMS) are significant predictors for Salzburg Stress Eating Scale (SSES) scores, *R*^2^=0.3048, F(8,1213)=67.92 *p< 0.005*.

**Table 6:** Predicting linear model SSES score

	Coefficient β* (SE)	t	***p***-value
**BMI**	0.194 (0.024)	7.777	*>0.005*
**BigFive_Agreeableness**	-0.051 (0.024)	-2.113	*0.035*
**TEMS_Habits**	0.105 (0.025)	4.263	*>0.005*
**TEMS_Hunger**	-0.065 (0.025)	-2.596	*0.010*
**TEMS_Health**	0.072 (0.026)	2.727	*0.006*
**TEMS_TradtionalEating**	-0.065 (0.025)	-2.616	*0.009*
**TEMS_WeightControl**	0.056 (0.026)	2.149	*0.032*
**TEMS_AffectRegulation**	0.448 (0.026)	0.026	*>0.005*

*z-standardized coefficients **β**

## Discussion

In this survey stress-overeaters were compared to non stress-overeaters (stress-undereaters and stress-insensitive eaters) regarding associations to (stress-related) behavioural characteristics and personality types. We were able to identify certain characteristics of stress-overeaters that need to be discussed in more detail.

Based on the validated SSES [21], we identified about 42% of participants as stress-overeaters and 58% as non stress-overeaters (among the latter 79% eating less, 21% eating equal). The results of the stress-eating self-assignment revealed that subjectively more participants rated themselves as stress-overeaters (48%). Objective and subjective stress-eating classifications were strongly related, indicating that most of the affected individuals are aware of their vulnerability to overeating in stressful situations. Similar stress-eating distributions were found by Oliver and Wardle [32], investigating a sample of 212 undergraduate students. They also identified 42% of their participants being hyperphagic in stressful situations, but only 38% to eat less under stress. Furthermore, our results disclose a positive association between stress-overeating and levels of perceived stress.

Regarding socio-economic and anthropometric characteristics, stress-overeating was significantly associated with sex and BMI. The latter is in line with Cotter and Kelly [33] examining cross-sectional data from 3,708 adults presenting a positive association between experienced stress and BMI as well as waist circumferences after controlling for age and other variables (2% variance).

Stress-eating can also be seen as a maladaptive response towards stress [34]. Therefore, participants’ stress-coping behaviour should be considered in relation to stress-eating. While most survey participants reported ‘Social Support’ as the most important coping strategy, none of the SCI strategies was found to be positively associated with stress-overeating. Wichianson et al. [35] investigated the link between perceived stress and night-eating syndrome in a sample of 95 undergraduate students. Their results revealed an association between maladaptive coping and unhealthy night-eating (β=0.25, *p<0.05*). Adequate coping was also found to be positively related to adherence to own dietary goals [36] and healthier food choices [37].

Besides coping behaviour, eating motivation was found to affect eating patterns. Our results demonstrate that the eating motives ‘Habits’ and ‘Weight Control‘ can predict stress-overeating. Regarding habits, it has been shown that the experience of stress activates habitual behaviour rather than goal-directed behavioural strategies [38]. In the context of this research, it can be interpreted that goal-orientated healthy eating behaviour is suppressed by unhealthy comfort eating habits to cope with stress. This is in line with findings by O’Connor and O’Connor [39] reporting about a group of 155 females trying to lose weight who increased the consumption of between-meal snacks in stressful situations. Additionally, persons identified as stress-overeaters had significantly higher importance scores of ‘Affect Regulation’ and ‘Weight Control’. These results are confirmed by Sproesser et al. [40] reporting a negative relation between ‘Affect Regulation’ and healthy eating patterns as well as an association between weight control and emotional eating on a sample of 761 females. Pool et al. [41] showed that the experience of stress provokes habitual eating patterns, which have long been established.

Stress not only influences the amount of food intake, but also food choices towards unhealthier food items. Our results demonstrate that chocolate, cookies, cake, ice cream, chips and crackers, salted nuts, sweets, as well as fast and fried foods were found to be positively associated with stress-overeating. This is in line with the findings by Macht and Mueller [42] depicting emotional eaters to have more intense chocolate cravings and accordingly a higher chocolate consumption. Similarly, Errisuriz et al. [43] found a positive association between perceived stress and the consumption of salty snacks and fast food as well as soda, coffee and energy drinks (*p<0.05*), in a sample of 613 students. In total, our results reveal that stress-overeaters tend to have a preference for snack-related food items, especially sweets. This is in agreement with a study of Oliver, Wardle and Gibson [44], showing in a sample of 68 healthy individuals that perceived stress is positively correlated with the consumption of less healthy foods, especially high-fat and energy-dense ones.

Regarding the link between stress and comfort foods, it should be taken into account that stress coping as well as dieting status were shown to have effects on the consumption of comfort foods. According to literature, individuals with a low ability to manage stress were found to have a greater consumption of sweet snacks [43]. Restrained eaters (i.e., dieters) tend to eat more when stressed, whereas non-restrained eaters (i.e., non-dieters) tend to eat less when stressed [32]. There is also a difference by sex. Women tend to be more vulnerable to stress-eating, increasing their food consumption when stressed [45, 46]. Our results also reveal a slightly higher stress-eating score (4.0%) for females compared to males.

Our study addressed the association between certain types of personality and stress-overeating. Out of the BigFive dimensions, only two dimensions were associated with stress-overeating. ‘Neuroticism’ was found to significantly differ between stress-overeaters and stress-insensitive eaters. This finding can be explained by the fact that individuals with high scoring in ‘Neuroticism’ tend to be insecure, nervous and depressed [27] and perceive stress more intensely than other types of personality [47]. Furthermore, Keller and Siegrist [48] reported a promoting effect of ‘Neuroticism’ for unfavourable food choices and overeating styles. ‘Agreeableness’, in contrast, was revealed as a negative predictor for stress-overeating. Recording to literature, high scores of ‘Agreeableness’ are assigned to persons with a high level of interpersonal trust and cooperation as well as altruistic behaviour [27]. In contrast, low levels of ‘Agreeableness’ are considered as a risk factor for the development of eating disorders [49]. A high level of ‘Conscientiousness’ was found to be associated with healthy dietary behaviour as well as better health in general [50]. Additionally, ‘Conscientiousness’ and ‘Openness’ are related to a higher intake of fruit and vegetables [49].

### Strengths and limitations

The present study dealt with a large variety of pre-selected predictors of stress-overeating. The selection of predictors was based on an intense literature search and validated questionnaires were used for the assessment of potential predictors. This paper can therefore only serve as a contribution for a better characterization of stress-overeaters, covering a broad spectrum, ranging from personality dimensions over coping strategies up to anthropometric measurements. However, we cannot claim that the complete set of predictors for stress-induced hyperphagia was considered and additional research is needed. Furthermore, the cross-sectional design only allows for conclusions on potential associations between parameters, but it does not provide proof of causal relationships. The examined large German sample is not representative for the general population. It mainly consisted of young female students, therefore results cannot be generalized and further research in other population groups is necessary. Besides, the survey was conducted within a period of time, where governmental restrictions due to the global COVID-pandemic were legislated in Germany. Possible effects of the pandemic might contribute to unconsidered influences on stress and eating behaviour in the context of different types of personality.

## Conclusions

The objective of this study was to identify characteristics of stress-overeaters. Taken together perceived stress, dietary behaviour aspects and certain personality dimensions seem to be relevant predictors for stress-overeating. Especially, ‘Agreeableness’ personality and eating motives ‘Hunger’ and ‘Traditional Eating’, are negatively associated with stress-overeating, while the eating motives ‘Habits’, ‘Health’ ‘Weight Control’ and ‘Affect Regulation’ positively predict stress-overeating. Stress-overeating was found to evoke unhealthy, especially snack-related sweet food choices, called ‘comfort foods’. This hyperphagic behaviour affects about 42% of the survey population. The identified characteristics could possibly help to better predict potentially vulnerable subgroups and to develop target group-specific interventions to support healthier dietary patterns in the context of stress.

## Data Availability

The datasets generated and/or analyzed during the current study are not publicly available due data security but are available from the corresponding author on reasonable request.

## Abbreviations

BFI-10: Big Five Inventory (10 items)
BMBF: German Ministry for Education and Research (Bundesministerium für Bildung und Forschung)
BMI: Body Mass Index
CRH: Corticotropin Releasing Hormone
DALY: Disability-Adjusted Life Years
HPA: Hypothalamic Pituitary Adrenal
PSS: Perceived Stress Scale
SCI: Stress and Coping Inventory
SSES: Salzburg Stress Eating Scale
TEMS: The Eating Motivation Survey
